# Low-Level Laser Therapy (904 nm) Counteracts Motor Deficit of Mice Hind Limb following Skeletal Muscle Injury Caused by Snakebite-Mimicking Intramuscular Venom Injection

**DOI:** 10.1371/journal.pone.0158980

**Published:** 2016-07-08

**Authors:** Willians Fernando Vieira, Bruno Kenzo-Kagawa, José Carlos Cogo, Vitor Baranauskas, Maria Alice da Cruz-Höfling

**Affiliations:** 1 Department of Semiconductors, Instruments and Photonics (DSIF)–Faculty of Electrical Engineering and Computation, State University of Campinas (UNICAMP), Campinas, São Paulo, Brazil; 2 Institute for Research and Development (IP&D)—University of Vale do Paraíba (UNIVAP), São José dos Campos, São Paulo, Brazil; 3 Department of Biochemistry and Tissue Biology (DBBT)–Institute of Biology, State University of Campinas (UNICAMP), Campinas, São Paulo, Brazil; Universidad de Costa Rica, COSTA RICA

## Abstract

Myotoxins present in *Bothrops* venom disrupt the sarcolemma of muscle fibers leading to the release of sarcoplasmic proteins and loss of muscle homeostasis. Myonecrosis and tissue anoxia induced by vascularization impairment can lead to amputation or motor functional deficit. The objective of this study was to investigate the dynamic behavior of motor function in mice subjected to injection of *Bothrops jararacussu* venom *(Bjssu)* and exposed to low-level laser therapy (LLLT). Male Swiss mice received *Bjssu* injection (830 μg/kg) into the medial portion of the right gastrocnemius muscle. Three hours later the injected region was irradiated with diode semiconductor Gallium Arsenide (GaAs– 904 nm, 4 J/cm²) laser following by irradiation at 24, 48 and 72 hours. Saline injection (0.9% NaCl) was used as control. Gait analysis was performed 24 hours before *Bjssu* injection and at every period post-*Bjssu* using CatWalk method. Data from spatiotemporal parameters Stand, Maximum Intensity, Swing, Swing Speed, Stride Length and Step Cycle were considered. The period of 3 hours post venom-induced injury was considered critical for all parameters evaluated in the right hindlimb. Differences (p<0.05) were concentrated in venom and venom + placebo laser groups during the 3 hours post-injury period, in which the values of stand of most animals were null. After this period, the gait characteristics were re-established for all parameters. The venom + laser group kept the values at 3 hours post-*Bjssu* equal to that at 24 hours before *Bjssu* injection indicating that the GaAs laser therapy improved spatially and temporally gait parameters at the critical injury period caused by *Bjssu*. This is the first study to analyze with cutting edge technology the gait functional deficits caused by snake envenoming and gait gains produced by GaAs laser irradiation. In this sense, the study fills a gap on the field of motor function after laser treatment following snake envenoming.

## Introduction

Estimated global incidence of snakebites amounts to more than 5 million cases every year, from which 400,000 people are left with permanent sequels and 25,000 to 125,000 die [1]. The World Health Organization considers snakebites envenoming a neglected tropical disease which needs an integrated global strategy for controlling the problem. Depending on the severity of accident, snakebites impose a health and economic burden to victims who are generally male young people living in low-income rural regions of tropical countries. The distance from public emergency medical service aggravates the problem. The largest number of snakebites in Latin America is attributed to lance-headed pit vipers (genus *Bothrops*, family *Viperidae*) [2,3]. In Brazil, *Bothrops* genus accounts for nearly 90% of accidents; although Bothropic envenoming shows low lethality (less than 0.5%), morbidity is high due to incidence of cases, period of immobilization and the severe set of fast local complications [4]. The proteolytic, coagulant, hemorrhagic and myotoxic activities of toxins contained in venom [5] cause at the bite site muscle necrosis, thrombosed blood vessels, ischemia and destruction of intramuscular nerve trunks which hampers or avert tissue regeneration [6]. Antivenom treatment, although effective against systemic effects, shows low or none effectiveness against such local pathology syndrome [7,8]. The search for alternative and high-impact interventions is needed to minimize the local effects of snakebites.

Numerous studies have reported the biostimulation effects of phototherapy in repair processes. The efficacy of low-level laser therapy (LLLT) relies on the ability of light radiation to stimulate biological processes [9–11]. Positive effects of photobiostimulation include mitosis activation and cell proliferation, modulation of cell differentiation, protein synthesis, increases in adenosine triphosphate (ATP) content and muscle contractile activity, activation of growth factors, interleukins and inflammatory cytokines and protection against free radicals formation [10,12–15]. Such effects elect laser therapy a good approach to minimize myonecrosis, accelerate satellite cells proliferation and restore innervation and combat inflammation caused by hemorrhagins, myotoxins, neurotoxins and metalloproteinases of Bothropic venoms. In fact, since the work by Dourado and colleagues [16] which first reported the benefits of LLLT to counteracting myonecrosis caused by *Bothrops moojeni* venom, several studies have validated these findings [17–19]. However, as far as the authors know none of them analyzed the motor functional activity in muscles injected with snake venom and exposed to low-level laser irradiation. The purpose of this study is to test the hypothesis that the reported benefits of LLLT are accompanied by improvement of the motor functional activity after venom intramuscular injection-mimicking the bite of the terrestrial species *Bothrops jararacussu* bite. Details of the pathological syndrome of victims are reported by Milani et al. (1997) [20].

Hitherto, the parameters that have been used for analysis of motor function included sciatic functional index (SFI) [21,22], static functional index (SSI) [23–25], posture factor [25], ankle kinematics [26], toe out angle (TOA) [27,28] or peroneal functional index (PFI) [23]. The CatWalk is a system able to assess the motor function dynamically in its entireness since it encompasses the majority of those parameters abovementioned [29]. Studies associated with venomous ophidian outcomes are of public health interest; this study adds novel data on the potential use of laser in skeletal muscle recovery.

## Materials and Methods

### Ethics statement and experimental groups

The experimental protocol was approved by the University’s Committee for Ethics in Animal Use (CEUA/UNICAMP, Protocol 2950–1) and was done in accordance with the general ethical guidelines of the Brazilian Society for Laboratory Animal Science (SBCAL) and the International Guiding Principles for Biomedical Research Involving Animals (CIOMS/ICLAS). Thirty male Swiss mice (*Mus musculus*), 6-8-week-old, weighing 22 ± 3 g were used. The animals were randomly selected into six groups (n = 5 per group): S- saline group, sterile saline solution (0.9%) injection; SL- sterile saline (0.9%) injection plus LLLT; SPL- saline injection plus laser not plugged, placebo group; V- venom group, injection of *Bothrops jararacussu* venom (*Bjssu*); VL- *Bjssu* plus LLLT and VPL- *Bjssu* plus laser off, venom placebo group.

*Bothrops jararacussu* lyophilized venom (*Bjssu*) was maintained at -20°C and dissolved in sterile 0.9% saline solution at the time of use. A single sub-lethal dose of *Bjssu* (830 μg/kg; volume of 20 μl) was intramuscularly (i.m.) injected into the medial portion of the right gastrocnemius muscle. After injection, the animals were placed in standard cages in a room with controlled temperature and humidity and received food and water *ad libitum*.

### LLLT protocol

LLLT was performed using a gallium-arsenide (GaAs) diode laser (BIOSET^®^ *Physiolux Dual*, Rio Claro, SP, Brazil), with parameters indicated in Table 1 [30]. The light was right at the site of venom or saline injection on the middle portion of the right gastrocnemius muscle (envenomed region). The timeline for laser irradiation was at 3, 24, 48 and 72 h after *Bjssu* injection; the laser parameters are shown in Table 1.

**Table 1:** Laser Parameters.

**Active laser**	**λ**	**Medium power**	**Spot area**	**Pulse time**
*GaAs*	*904 nm*	*25 W*	*0*.*2 cm*	*200 ns*
**Emission mode**	**Peak power**	**Energy density**	**Time**	**Frequency**
*Pulsed*	*7 W*	*4 J/cm²*	*1 min 32 s*	*2075 Hz*

Parameters were chosen based in the recommendations of the World Association for Laser Therapy (WALT); λ = wavelength.

### Gait analysis

**Fig 1:**
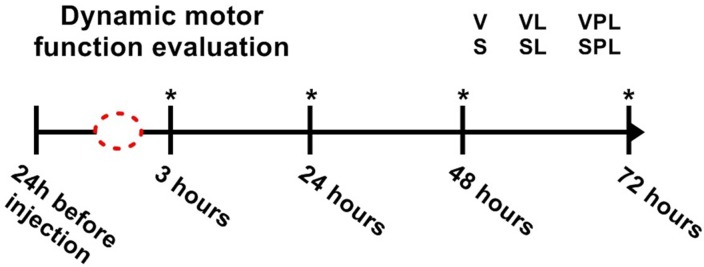
Dynamic motor function evaluation. Data from animals were measured 24 h before (baseline) and 3, 24, 48 and 72 h after venom or saline injection. Groups: V (venom); VL (venom + laser); VPL (venom + placebo laser); S (saline); SL (saline + laser); SPL (saline + placebo laser). *GaAs laser irradiation periods or laser simulation (sham or placebo group); *Bjssu* or saline injection = dotted red circle.

The parameters to analyzing the motor activity were assessed using the CatWalk XT^TM^ system (Noldus Information Technology^®^ - Netherlands). Measurements of mice motor activity were evaluated 24 h before saline or venom injection (baseline data) and then at 3, 24, 48 and 72 h after injection and GaAs laser irradiation at the timeline scheduled (a fake irradiation was also performed, which was designed placebo laser) (Fig 1). A digital camera located under the walkway was set at 30.99 gain and 0.15 intensity threshold. The mice were released from one side of the walkway to spontaneously achieve the opposite side. At each time, each animal of the group performed 15 runs, but the data of each run were recorded just when the animal was able to cross a calibrated 20 x 10 cm length lane and spending a minimum of 0.5 sec and maximum of 8 sec at a maximum speed variation of 40%. For each animal, the amount of recorded runs was n = 3, hence each experimental group amounted fifteen runs for each period (n = 5 animals/time). After the last period (72 h) of CatWalk system XT^TM^ 9.1 software recordings, the animals were euthanized in a CO_2_ chamber followed by cervical dislocation.

**Fig 2:**
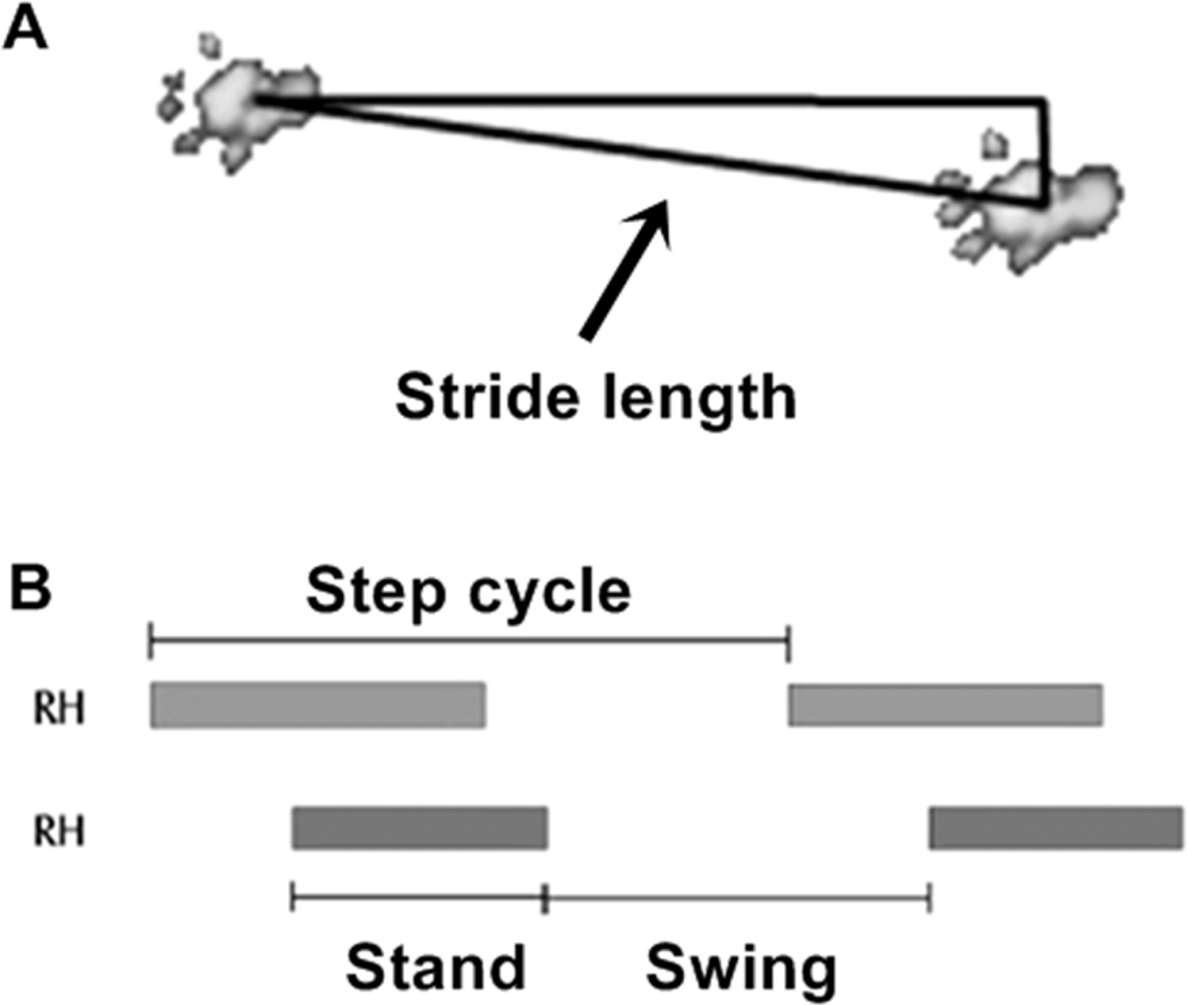
Stride Length and Step Cycle. (A) Representative scheme of stride length related to distance between two successive placements of the same foot. (B) Representative scheme of step cycle parameter, that is equal to stand + swing. RH = right hindlimb. Bars represent the stand, while empty spaces represent the swing phase.

The parameters computer-generated by CatWalk XT^TM^ 9.1 software following the image capture of the runs produced graphs of paw pressure and a list of static and dynamic records related to single paws, as follows:

- *Stand*: duration (in seconds) of mice paw contact with the glass plate;
- *Maximum Intensity*: maximum intensity (in arbitrary units) referring to pressure exerted by the paws, considering the mean of full track;
- *Swing*: time duration (in seconds) without mice paw contact with the glass platform;
- *Swing Speed*: paw speed (in centimeters/second) during the swing;
- *Stride Length*: distance (in centimeters) between two placements of the same foot. The calculation of this gait parameter is based on the X-coordinates of two successive footprints of the same foot for maximum contact center positions, taking into account the Pythagorean Theorem (Fig 2A).
- *Step Cycle*: time (in seconds) between two consecutive initial contacts of the same foot (Fig 2B).

### Statistical Analysis

Each parameter of dynamic motor function was analyzed by *one-way ANOVA* followed by *Tukey's Multiple Comparison* test or by Student *t*-test and expressed as mean ± standard deviation (SD). The significance level was set at *p<0.05.

## Results

No complication after *Bjssu* injection (830 μg/kg) in the mice was noted. All 30 animals stayed alive until the end of experiment.

### Effect of LLLT on the general walking pattern

**Fig 3:**
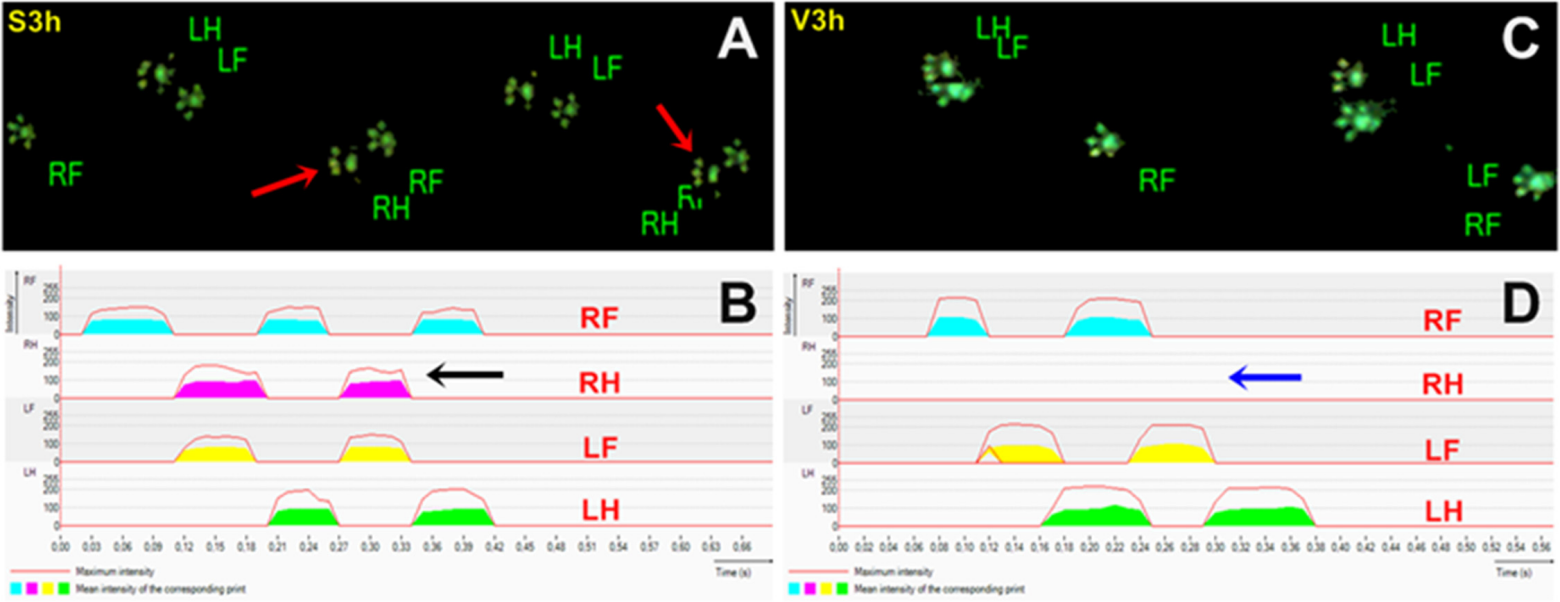
General walking pattern: 2D representative schemes. Gait patterns registered 3 h after the mice had received i.m. injection of saline or *B*. *jararacussu* snake venom into the right gastrocnemius. (A) Mice exhibited a normal gait pattern, with paw imprints of the saline injected right hindlimb (RH, red arrows) perfectly delineated on the captured image; (B) Respective 2D footprint graphic representing maximum intensity of the right hindlimb (RH) footprint register (black arrow), as well as were also normally imprinted the right frontlimb (RF), left hindlimb (LH) and right frontlimb (LF), thus indicating that at 3 hours after injection/manipulation an even RH gait was preserved at every run. (C) RH footprint was absent in the lane what was confirmed by the 2D software footprint register (D), thus indicating that mice show zero intensity of RH footstep 3 hours after *Bjssu* injection into the right gastrocnemius (blue arrow). Notice that the intensity of the footstep of LH, LF and RF is higher, since the area of the pad and fingers appears strongly delineated relative to what is seen in the images of animals injected with saline.

The direct visual analyses of gait videos showed that the right and left paw imprints of S, SL and SPL groups showed no alteration in all parameters registered at 3 h, thus indicating a normal walk during footpath. On the contrary, 3 h after *Bjssu* injection (V-3 h and VPL-3 h groups) animals i.m.-injected with *Bjssu* and which were not submitted to LLLT cannot put weight on the right hindlimb (RH) during the runs, thus indicating hindrance of motor activity (Fig 3).

**Fig 4:**
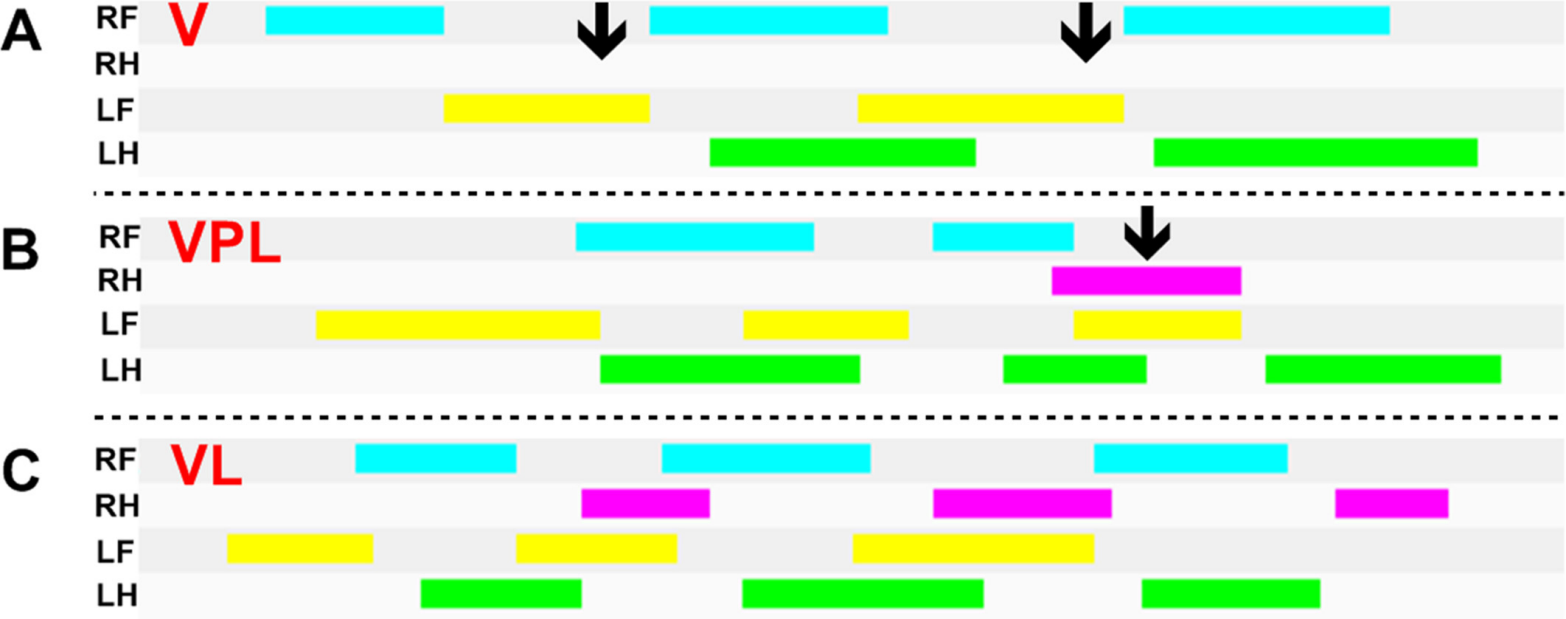
Timing diagrams: duration of pathway contact for a given paw in V, VPL and VL groups. (A) Representative illustration of mice feet prints registered during walking by the CatWalk system at 3 hours after *Bothrops jararacussu* venom (*Bjssu*) injection, (B) *Bjssu* injection treated with placebo laser and (C) *Bjssu* injection followed by GaAs laser irradiation. The paw-pathway contacts for each of the four paws over time are indicated. The length of each bar represents the duration of the stand phase for that particular paw. The space between bars represents the duration of the swing phase. The absence of the stand phase (A) as well its delay (B) in the use of the injured limb (RH—pink bars) is observed in V and VPL groups (arrows). The panel (C) shows a pattern of VL feet prints, which did not differ significantly from the baseline print registered 24 h before *Bjssu* nor from the S, SL and SPL prints (not shown), and hence consistent with a normal gait. RF = right frontlimb, RH = right hindlimb, LF = left frontlimb, LH = left hindlimb.

The animals of the VL-3 h group which had been irradiated with GaAs semi-conductor laser showed a normal gait as expressed by the paw imprints which did not significantly differ neither from the baseline paw imprints nor from the imprints of S, SL and SPL rats at the same time interval (Fig 4).

**Fig 5:**
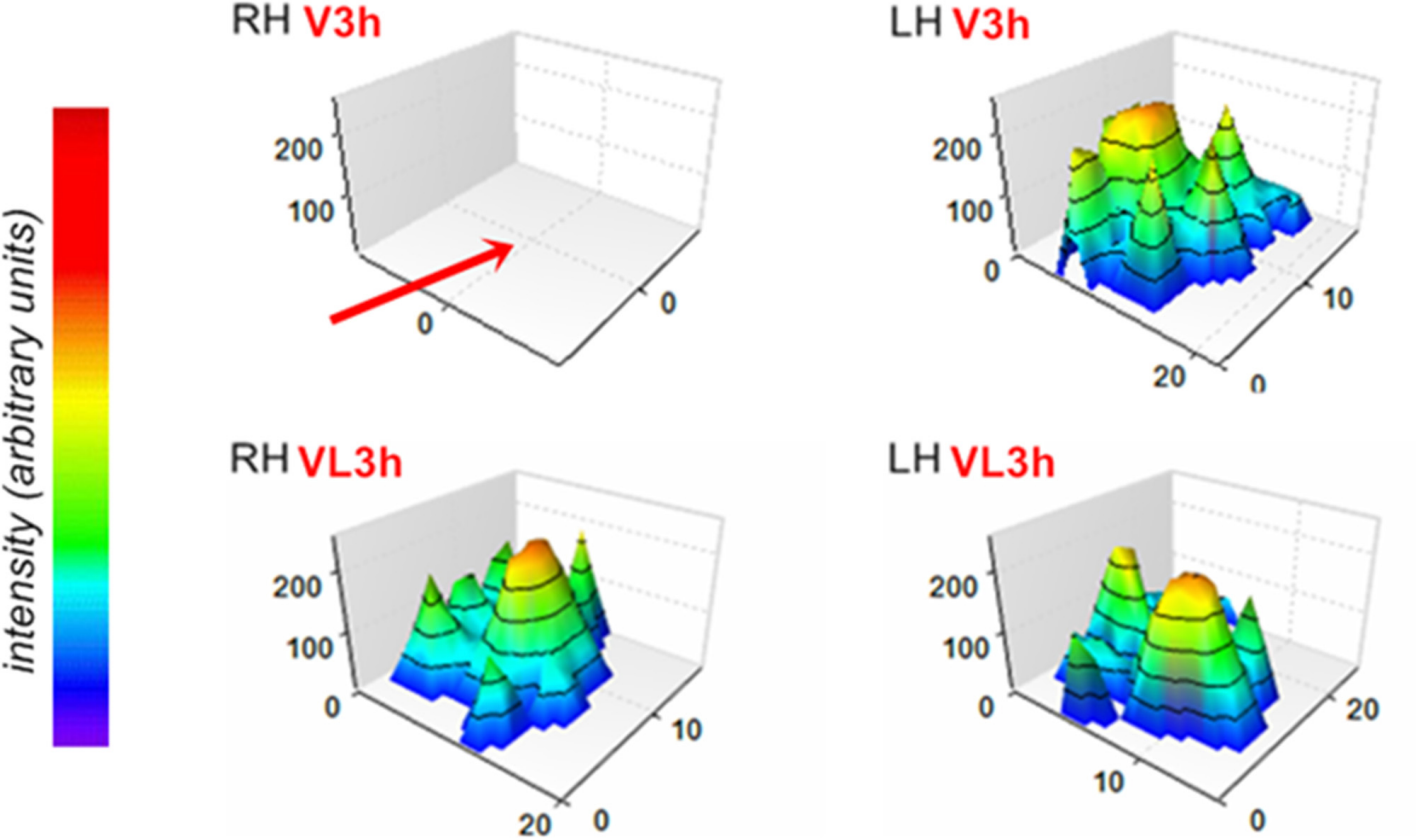
Intensity according pressure performed by the paws. 3D graphics showing zero pressure intensity for the right hindlimb (RH–red arrow) and the relative intensity of the fingers of LH (left hindlimb), characterizing the gait pattern mostly related to group V animals (and VLP not shown) at 3 h after *Bjssu* injection (upper left and right graphics). The lower, left and right graphics illustrate the normal pressure intensity of the RH and LH of mice that had been i.m.-injected with *Bjssu* and submitted to LLLT, hence characterizing regular gait pattern of VL-3 h mice, and which did not differ from the observed in the S-, SL- and SPL-3 h groups or 24 h before *Bjssu* injection (not shown).

Three-dimensional graphics for the right hindlimb generated from pressure applied by the right paws on the footpath remained at zero arbitrary units for V and VPL groups at 3 hours post-*Bjssu* (Fig 5). For the mice of the VL group the intensity of treading was captured without visually significant changes relative to S-, SL- and SPL-3 h groups or 24 h before *Bjssu* i.m.-injection (not shown). It is noteworthy that there is difference in the pressure in the left paw of contralateral hindlimb (LH); however, the statistical analysis showed no difference relative to the other periods/groups.

### Parameters related to single paws

The GaAs laser irradiation (904 nm; 4 J/cm^2^ for 92 sec) was able to improve most of gait parameters in the acute 3 h period following envenomation. The period of 3 h following *Bjssu* injection was shown to be critical for all parameters evaluated for the RH of mice from venom (V) and venom-placebo laser (VPL) groups. At this time all the parameters of the mice gait were statistically different from the baseline level observed in the groups S, SL and SPL and including in the VL group, whose venom effect was prevented by the phototherapy promoted by LLLT. The other periods, 24, 48 and 72 h showed parameters statistically not different from those observed 24 h before *Bjssu* exposure (and also from the steady or baseline parameters).

#### Stand and Maximum Intensity

The duration of mice paw stance on the glass printing plate significantly decreased in the animals of V-3 h group indicating important motor deficit during gait (*p<0.05). In contrast, in the VL-3 h group the motor deficit was prevented by GaAs laser irradiation at the site of *Bjssu* injection in the gastrocnemius of the right hindlimb. As result the stand of VL-3 h mice did not differ statistically from S-, SL- and SPL-3 h mice, or in either of the periods including to the registered 24 h before *Bjssu* i.m. exposure. The data thus indicate that as time post-*Bjssu* injection elapses the motor activity is being restored to normality; the findings also revealed that the injection of saline was harmless to the motor activity of S, SL and SPL groups.

**Fig 6:**
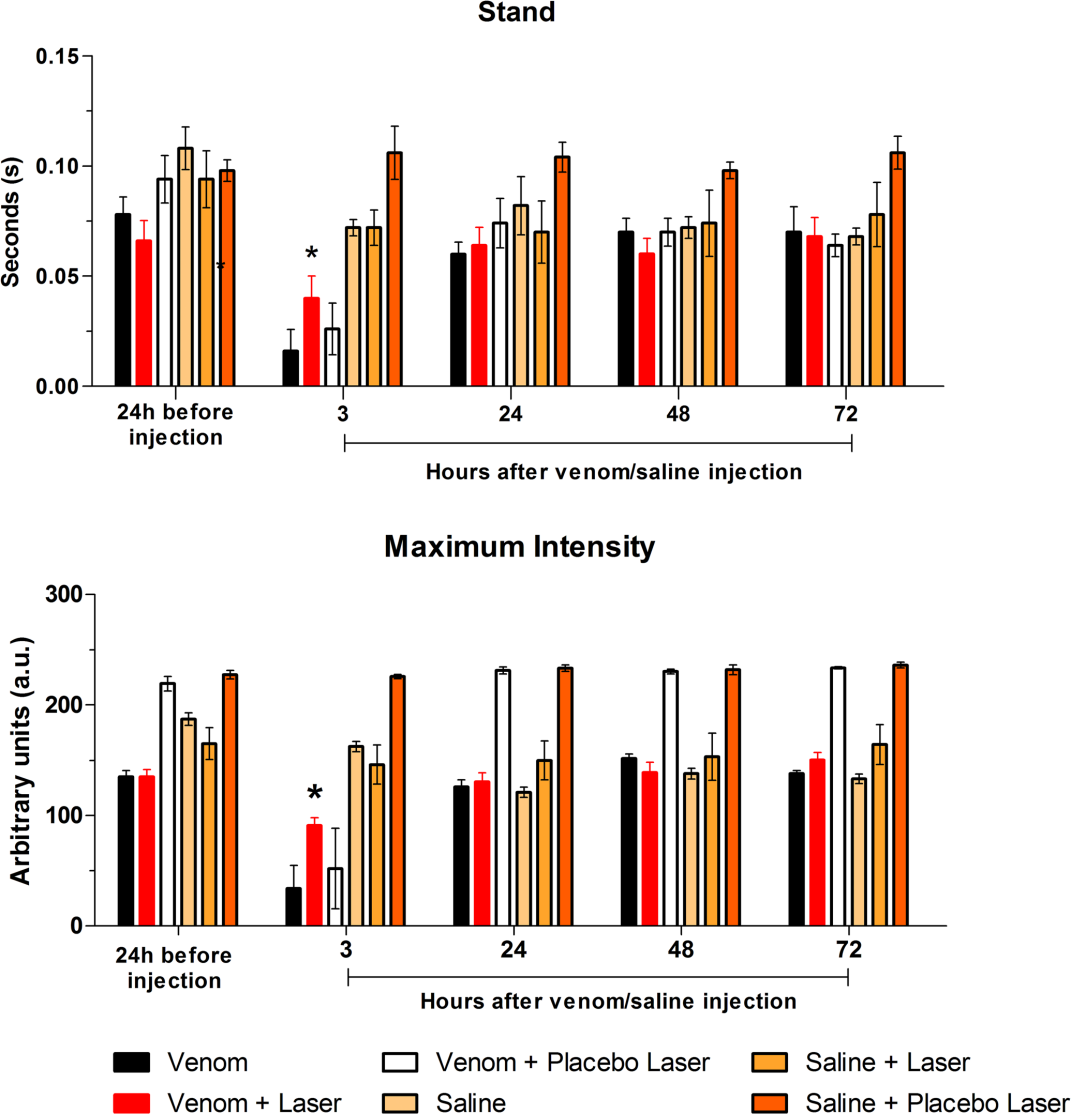
Stand and Maximum Intensity parameters evaluated in the right paw. Three hours following *Bjssu* injection both parameters worsened in animals whose right gastrocnemius was injected with venom (V group) and was sham-irradiated (VPL group). The laser-treated venom group (VL) showed no significant difference compared to baseline and the post *Bjssu* injection periods. Data are expressed as mean ± SD; One-way ANOVA followed by Tukey's Multiple Comparison test; *p<0.05 compared to V and VPL at the same time-point of 3 h; #p<0.05 compared to other interval within the V or VPL groups.

Also, there was no difference in the stand parameter between groups V-3 h and VPL-3 h (laser sham) since 3 h post-*Bjssu* injection, three animals out of 5 of both groups did not touch the footpath platform, while the other two stand the right paw just for 0.04 seconds over the platform. Also, there was no differences between groups V and VPL in either of time intervals; nonetheless, as the time goes by the duration of mice paw contact with the glass printing plate (stand) increased causing significant differences intra-V and–VPL groups (#p<0.05; Fig 6). The data indicate an important motor deficit at acute stages of envenoming corroborating morphological studies that show intense myonecrosis fast developed soon after (*Bothrops*) venom inoculation [16].

Three out of five animals that were injected with *Bjssu* (V-group) failed in performing the Maximum Intensity with the right paw all the way through the walkway 3 h post-venom. Opposed to this, animals of the VL group whose right gastrocnemius had been injected with *Bjssu* and subsequently were submitted to GaAs LLLT showed that the mean of the Maximum Intensity of the right paw remained above zero 3 h post-*Bjssu* all through the entire track. The Maximum Intensity values of VL-3 h group was significantly below the baseline values found 24 h before venom injection (92±15.2 vs 135±14.1) and 72 h (92±15.2 vs 150.3±14.8) (p*<0.05), but significantly above the exhibited by rats from group envenomed but not submitted to LLLT (V-3 h and VPL-3 h). The findings suggest that the laser treatment applied soon after the *Bjssu* administration was efficient in probably preventing pain and edema formation (Fig 6). The Maximum Intensity displayed by the right paw of V-3 h and VPL-3 h differed significantly (#p<0.05) within the same group relative to the other intervals. The data confirmed the 3 h post-*Bjssu* as critical for the parameters evaluated.

#### Swing Speed and Swing

**Fig 7:**
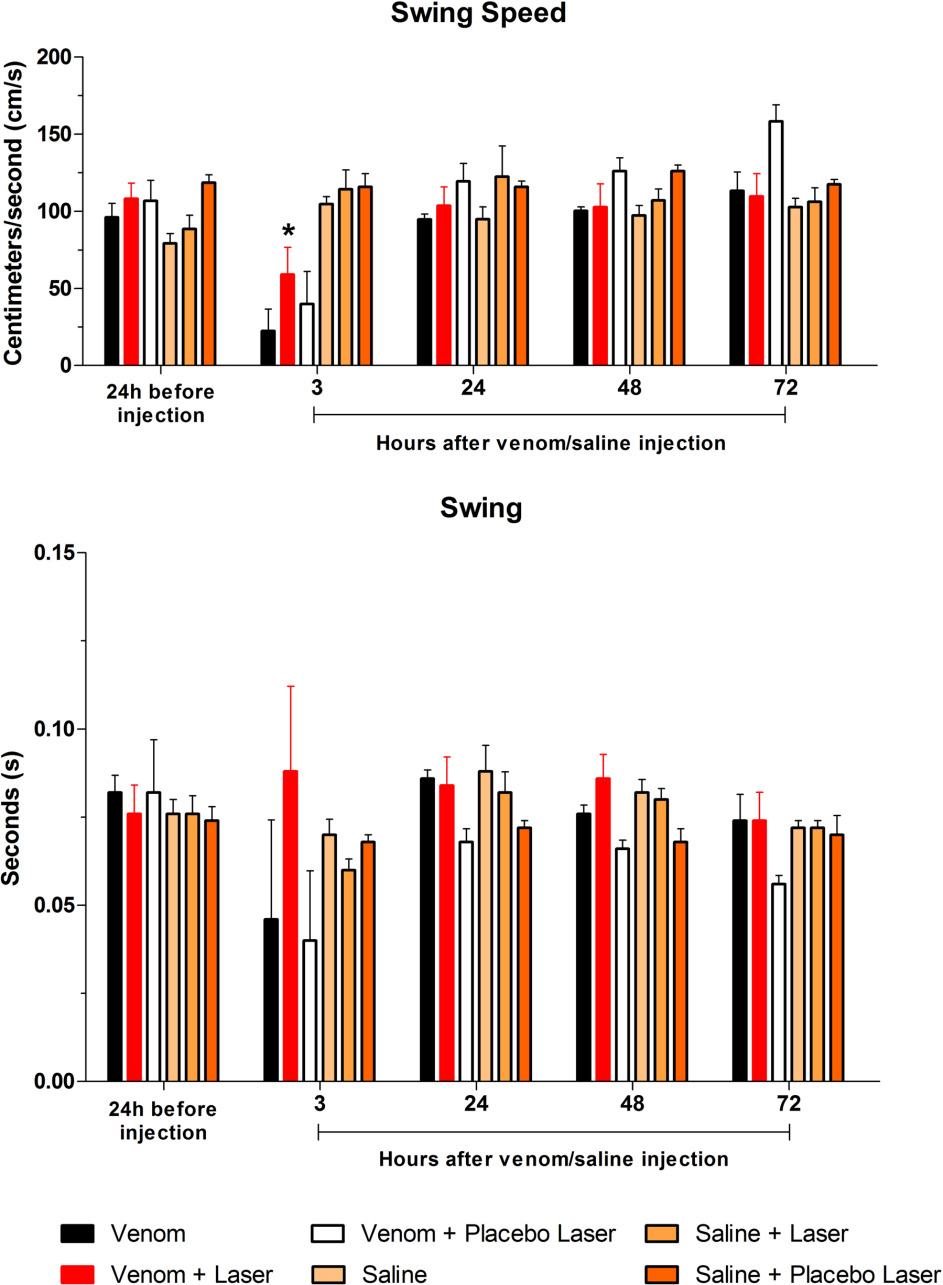
Bar graphs showing Swing Speed and Swing. In both parameters difference (p*<0.05) was observed at 3h post-injection, mean for Swing Speed of V and VPL groups. The laser treated group (VL–red bar) showed no significant difference compared to baseline and the post *Bjssu* injection periods for the Swing parameter. Data are expressed as mean +/- SD; statistical analysis were performed by One-way ANOVA followed by Tukey's Multiple Comparison test; (*) when the group VL is different from V and VPL (p*<0.05) in the same period.

For the Swing Speed, which measures the paw speed during the swing, the GaAs laser was able to accelerate the elevation of the right hindlimb in VL-3 h, compared to V-3 h and VPL-3 h (*p<0.05), which had been impaired relative to baseline, i.e. the value at 24 h before injection. There was significant differences in V-3 h and VPL-3 h within each group relative to the other time-points (#p<0.05, Fig 7). The data corroborate that at acute stages post-exposure of gastrocnemius to venom, there was shortage of the motor activity and that pain/edema plus tissue damage had a role in the motor deficit. For the Swing, interval during which mice did not contact its right paw on the glass platform, significant difference (*p<0.05) was observed only in the SL group when comparing 3 h vs 24 h (0.06±0.01 vs 0.08±0.01) and 3 h vs 48 h (0.06±0.01 vs 0.08±0.00) periods. The GaAs laser treatment showed no significant difference compared to baseline and the post *Bjssu* injection periods (see discussion).

#### Stride Length and Step Cycle

**Fig 8:**
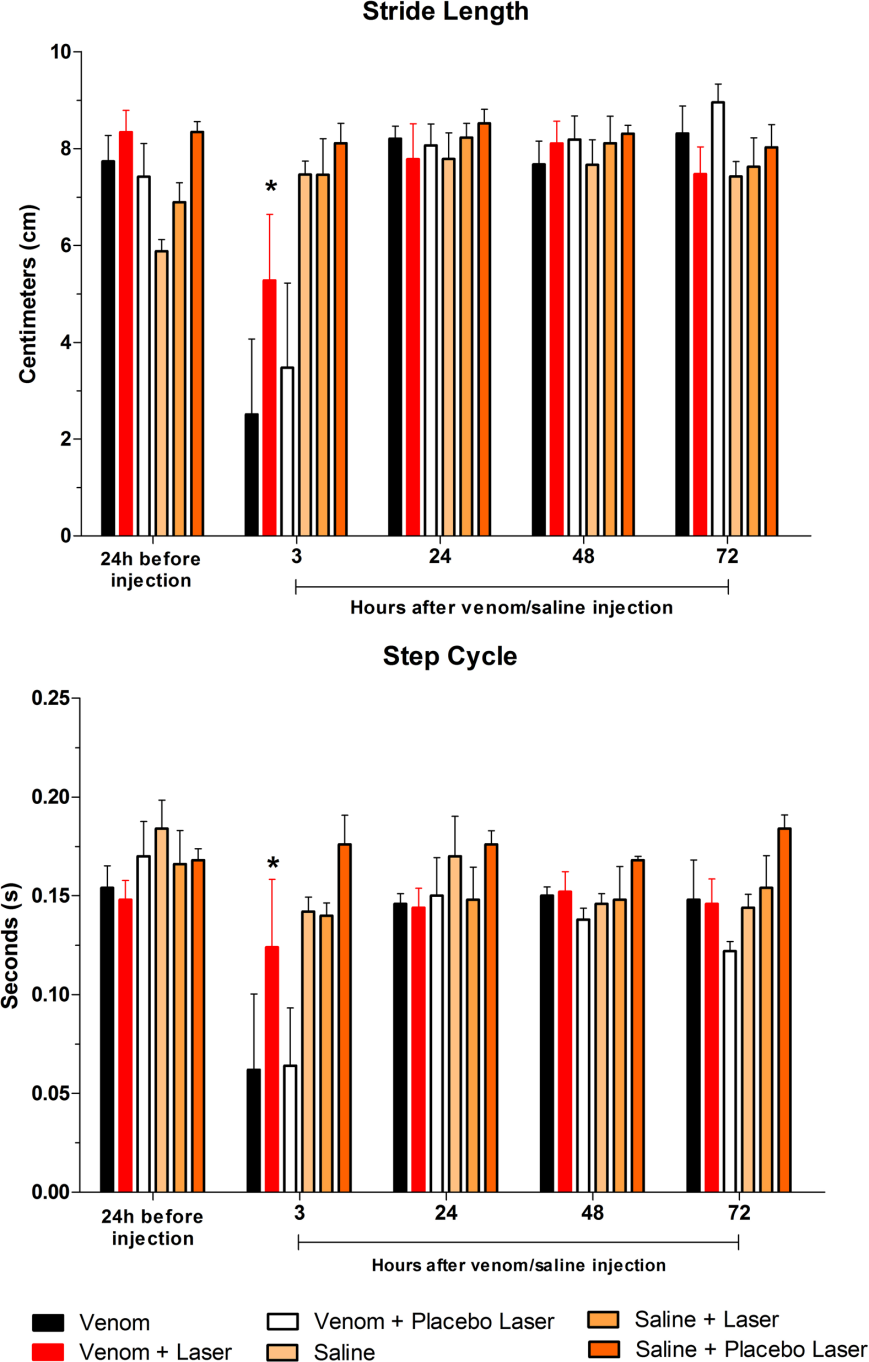
Bar graphs showing Stride Length and Step Cycle. The period of 3 hours following *Bjssu* injection was critical for V and VPL groups, like in the previous parameters. The laser treated group (VL–red bar) showed no difference compared to baseline and post-injection periods for both parameters, but was different from V-3 h and VPL-3 h in the same period. Data are expressed as mean +/- SD; statistical analysis were performed by One-way ANOVA followed by Tukey's Multiple Comparison test; (*) when the group VL is different from V and VPL (p*<0.05) in the same period.

The distance (in centimeter) between two placements of the very same right paw on the platform showed no difference for group VL-3 h comparing with baseline and post *Bjssu*/saline injection. The GaAs laser irradiation improved the Stride Length when comparing to groups V-3 h and VPL-3 h in the same period. Significant difference (*p<0.05) was observed in the V and VPL groups when comparing 3 h and the other intervals in the same group (Fig 8). Since the animals of the groups V-3 h and VPL-3 h stand the paw of the right hindlimb over the platform for a few fraction seconds (± 0.04s), the Stride Length was shorter (see Figs 3D, 4A and 5). Like in Stride Length, the Step Cycle (the time in seconds between two consecutive initial contacts of the same paw with the glass platform) of group VL-3 h showed no difference between pre and post venom/saline injection periods. Thus, it indicates that the GaAs laser irradiation was also able to improve the Step Cycle, comparing with V-3 h and VPL-3 h groups. Likewise the observed in the previous parameters, significant difference (*p<0.05) in the V and VPL groups was shown when comparing with other intervals within the same group (Fig 8). The VL, S, SL and SPL groups showed no difference compared to each other, for all post-injection periods. Therefore, it is inferred that the laser irradiation brought benefits both spatially (Stride Length) and temporally (Step Cycle).

## Discussion

Bites by venomous *Bothrops* snakes cause severe morbidity at the bite site, sometimes leading to permanent disability or even amputation of the member affected [2–6]. Local effects are of fast evolution. Phospholipase A_2_ (PLA_2_) present in the *Bothrops* venom disrupts the sarcolemma of muscle fibers leading to the release of sarcoplasmic proteins and loss of muscle homeostasis [31] soon after venom contact. The influx of Ca^2+^ through the disrupted fiber membrane triggers the catabolic activity of calcium-dependent endogenous proteases resulting in the proteolysis of myofilaments [32,33]. Muscle necrosis is the primary effect of *B*. *jararacussu* venom but vascular thrombosis, ischemia and further fibrosis at the site of muscle fiber losses contribute to hinder regeneration [5]. As result, functional deficits of the affected limb can compromise the standard gait characteristics.

There is evidence that biostimulation by low-level laser therapy (LLLT) increases cell proliferation, collagen synthesis and activation of growth factors [34,35]. The LLLT applied during the first hours after injury optimizes repair process of muscle tissue by increasing proliferation of satellite cells, mobility of myoblasts and growth of young myofibrils concomitantly with the formation of new blood vessels at the irradiated area [36–39]. The benefits of photobiostimulation promoted by low energy laser irradiation to minimize muscle inflammation, myofibers losses caused by venom myotoxins and improved regeneration have been already experimentally demonstrated [16–19]. However, no study evaluated if the motor function of the affected limb would be likewise benefited.

Methods destined to evaluate the efficacy of a therapeutic procedure in improving motor function in humans is of hard assessment due to the wide variability in the level of injury, the condition of the patient and differences in therapy parameters used. Animal models allow a better evaluation because there is a more controllable level of injury and parameters reproducibility for analysis.

Evaluation of functional recovery has been commonly used for assessing peripheral nerve lesions by using the *Sciatic Functional Index* (SFI) created by de Medinaceli and collaborators in 1982 [21]. This index consists of a quantitative system to assessing the sciatic nerve recovery and is widely used in cases like peripheral nerve lesions or neuronal disorders [21–27].

However, evaluation of functional recovery for assessing motor deficits generated by muscle injury is less available [22,40]. So far, studies with the CatWalk method have shown its employability in the analysis of motor function in models of pain [41,42], sciatic nerve injury [25,43] and induced osteoarthritis [44,45].

Herein, we took advantage of the sensitivity of the CatWalk method to assess gait parameters in mice whose right gastrocnemius had been injected with the venom of *B*. *jararacussu* and submitted to LLLT. To the best of our knowledge, this is not only the first work seeking to apply comprehensive gait analysis in animals undergoing myonecrosis caused by snake venom, but also pioneered the use of the CatWalk method for assessing the dynamic motor function over the time post-envenomation. In addition, records on the use of CatWalk system for analysis of the influence of LLLT on dynamic motor function apparently were never done.

The benefits of LLLT photobiostimulation on motor function had been described in models of injury and regeneration of peripheral nervous tissue. The therapy consisted in using GaAlAs (gallium-aluminum-arsenide) laser (830 nm; 100 mW; 140 J/cm²) irradiation for 21 consecutive days on the common peroneal nerve in rats submitted to crush injury, with treatment initiated on the first day post-injury [46]. The authors evaluated the gait through and the time spent to cross the walkway and determined the *peroneal functional index* (PFI) captured during the track. The crossing time and the PFI were significantly improved in the group of rats subjected to LLLT indicating that the treatment improved gait and accelerated regeneration of the peroneal nerve in rats.

In the present study, the CatWalk demonstrated to be an effective method for detecting positive motor effects of the GaAs laser irradiation after muscle envenomation by *B*. *jararacussu* venom. The computer-assisted register of the different parameters of mice footpath on the glass platform of the CatWalk system showed that deficits in motor activity were temporary. The critical period was 3 hours after *Bjssu* intramuscular injection. The most affected parameters were clearly Stand and Maximum Intensity. The laser irradiation improved all the gait parameters at the acute period (3 hours) after injection of *Bjssu* compared to baseline.

Studies have shown that intact control rats have “paw elevation time” around 10 seconds while in rats experiencing pain this interval increases because the rats hind paw fails to touch the surface of a rotating cylinder for 60 seconds (paw elevation time) [47]. The rotating cylinder was used also for evaluating the motor function of rats that had the gastrocnemius injected with 5% formalin and were further irradiated with GaAlAs laser (830 nm; 100 mW; 20, 50, and 100 J/cm²); the “paw elevation time” was measured before formalin injection (intact control) and 5 min, 2, 8 and 24 hours post-injury [48]. The authors found that the paw elevation time was higher at all post-injury periods compared to control meaning that the time post-injury and different laser density energies used affected equally the “paw elevation time” (named Swing parameter in the CatWalk system) since there was no difference between non-irradiated non-injected control and the gastrocnemius irradiated with 20, 50, and 100 J/cm² density energies. According to Grillner [49] swing time has traditionally been considered a constant parameter across all speeds in quadrupeds, characterizing the intrinsic dynamics of gait. This is in line with our data which showed no difference in Swing parameter all through the intervals in all examined groups (S, SL, SPL, V, VL and VPL) and even intra-same group at different intervals. Our results showed that while the velocity (including Swing Speed) and Stand changes over time, the Swing post-*Bjssu* or post-saline injection remain unchanged for all the groups (laser treated or not) compared with 24 h before injection (baseline value of intact control). Interestingly, the data show that the CatWalk sensitivity was able to capture and measure these particularities of gait that were not altered in the present snake envenoming model with or without GaAs laser treatment.

In normal human or animal gait, the absolute time of floor contact during a step will vary with velocity, respecting the fact that the time of single limb support from one limb must be equal to the Swing time of the opposite limb. Thus, the relative contribution from each foot at the same velocity remains the same. A faster gait cadence is achieved by putting more plantar flexion force at the ankle, i.e., the transfer of weight from heel to toe needs to be necessarily faster [26]. In our study, from the absence of stance phase of gait for the same right limb, all others parameters showed variation in the early hours at post-injury period. However, for the contralateral limb (left) there was no significant difference in the values of Speed and Maximum Intensity (data not shown), which could serve as a good indicator of a possible compensatory effect on the control (left) member.

Gastrocnemius muscle is extremely important in the stance phase of gait. It is a key structure in biomechanics that works even in extreme physical activity, with great risk of injury and rupture [40,50]. The gastrocnemius reflex-contraction takes the right hindlimb to remain on triple-bending. In the case of our animals this condition was reflected in the elevation of the limb. Our results showed a clear relationship with human epidemiological reports on venomous snakebites which describe loss of motor function in the first hours after envenomation [20]. The data corroborate with morphological studies that show intense myonecrosis which develops rapidly soon after (*Bothrops*) venom injection [6,31]. We suggest that triple-flexion shown by mice without treatment (V and VPL groups) may be possibly related to the pain indirectly caused by both the venom injection and motor nerve endings pressure induced by edema, or directly by degeneration of motor nerve fibers themselves caused by venom [6,8].

Generally, the painful condition related to the hindlimbs results in gait failure and/or gait inability, which is interpreted as a mechanism of protection triggered against aggravation of painful condition. On the other hand, chronic pain can cause muscle atrophy, disuse and habits of conscious or unconscious protection that can lead to severe loss of muscle function [48].

Currently, muscle function is also estimated by the maximum muscle fibers strength, measured during the process of recovery from muscle damage. Studies have shown that isometric force decreases immediately but transiently after the injury, followed by recovering over time. Generally the causes of functional loss related to muscle damage are studied by histologic evaluation. However, little information is available about the relationship between morphological and functional recovery [51,52]. According to Iwata et al., functional recovery of skeletal muscle occurs prior to the structural regeneration, while 90% of the muscular strength is required to restore normal locomotion [40]. In our study we did not perform tests for evaluating the muscle strength, however, the animals injected with *Bjssu* and remained untreated (V and VPL groups) had their function restored 24 h after induction of myonecrosis. The finding substantiate the idea that the return of function precedes normalization of muscle structure, which still appeared affected in the period of 72 h post-injury (own unpublished data). Thus we can also infer that LLLT accelerated the return of muscle function at the very early stages of post-injury period.

All of the gait parameters that we analyzed through the CatWalk method were sensitive to changes caused by myonecrosis of the gastrocnemius muscle, with focus in the period of 3 h post-injury. The S, SL and SPL groups were included in the analysis to observe if the injection (promoted by needle drilling itself) would cause any harmful effect—probably painful—to interfere in any of the parameters evaluated in motor function. The results correlated with the morphological analysis, which showed no changes in muscle tissue by injection of 0.9% NaCl differently from the observed in venom-administrated group (data not shown).

## Conclusions

Envenoming by snakebites is considered a neglected case of public health in tropical and developing countries, such as Brazil. No available treatment is currently effective against myonecrosis generated by this type of venom, so the idea of using LLLT is based on the fact that this device uses non-ionizing electromagnetic radiation of low intensity which is able to produce beneficial effects on tissues, stimulating the healing thereof. In this work, GaAs laser irradiation was able to improve spatially and temporally the gait parameters 3 hours after venom intramuscular injection-mimicking the terrestrial species *Bothrops jararacussu* bite. Three hours interval is critical in terms of destruction of muscle and nerve fibers and anoxia caused by perfusion failure caused by myotoxins and hemorrhagins present in venom. The high sensitivity of the motor dynamic function analysis by the CatWalk method was able to measure the gait deficit produced by snake venom and the benefits of the laser irradiation. All gait parameters were described in detail and could represent a paradigm for future works using the CatWalk method as a reference in the study of motor function in similar types of muscle injury. The LLLT is a resource used widely by therapists around the world, which ensures the beneficial and unexplored effect of this therapy. The fact that the technique is non-invasive and laser apparatus is easy to manipulate endorses LLLT as a technology to be explored in preclinical studies before recommended for clinical therapy in case of snakebites accidents. This experimental study fills a gap in the knowledge of motor function deficit resulting from snake envenoming before and after low-level laser treatment.
